# Ovarian ageing and the impact on female fertility

**DOI:** 10.12688/f1000research.16509.1

**Published:** 2018-11-22

**Authors:** Beverley Vollenhoven, Sarah Hunt

**Affiliations:** 1 Monash Health, Clayton, VIC, Australia; 2 Department of Obstetrics and Gynaecology, Monash University, Clayton, VIC, Australia; 3 Monash IVF, Clayton, VIC, Australia

**Keywords:** Age, oocyte, assisted reproductive technologies, fertility

## Abstract

Female fertility decreases with increasing age, a reflection of declining oocyte quantity and quality. The menopausal transition occurs when the oocyte quantity falls below a threshold level. The pattern of follicular depletion as well as the factors, timing and mechanisms surrounding both declining oocyte number and oocyte quality remain incompletely understood. Further studies are needed to examine the factors involved and develop predictive models and biomarkers to assist in the management of age-related subfertility. This review summarises the current knowledge addressing the ageing ovary and its impact on fertility.

## Introduction

Undisputedly, the single most important factor in determining fertility potential in the female is age. At a societal level, there has been a shift toward delayed childbearing. In Australia, the average age of women at first birth in 2015 was 28.9 years compared with 28.1 years in 2005 and the proportion of births to mothers over the age of 35 years was 22% ^1^. This in part has led to increased demand for reproductive assistance. The average age of women seeking assisted reproductive technologies in 2016 was 35.8 years as compared with 35.6 years in 2006. Over this 10-year time period, the age of women over 40 years undergoing an autologous *in vitro* fertilisation cycle rose from 1 in 5 to 1 in 4 ^2, 3^. Therefore, it is important to consider the regulators and physiological mechanisms involved in reproductive ageing.

It is well established that the female foetus possesses her entire quotient of primordial germ cells with a decline initiated and continued from about 16 to 20 weeks of pregnancy ^4^. There is no further neo-oogenesis. Follicular loss occurs via ovulation; however, the vast majority of follicles will undergo atresia before reaching ovulation. While preantral and pre-ovulatory follicles rarely undergo atresia, most follicular degeneration occurs at the follicle-stimulating hormone (FSH)-dependent, early antral follicle stage ^5^. The point at which menopause occurs corresponds to a decline in the primordial follicle pool below a critical threshold of about 750 to 1000 remaining follicles ^6– 9^. The loss of the primordial follicle pool is mirrored by a gradual loss in natural fertility over the reproductive lifespan followed by accelerated decline in the 10 years preceding menopause, reflecting quantitative and qualitative change to the ovarian oocyte reserve ^10^.

Previous modelling proposed a binary or biphasic pattern of follicular depletion with age. The binary model for follicular atresia hypothesises that follicular atresia occurs at two different rates over the reproductive lifespan. At a critical time point, previously accepted as approximately 37.5 years, the rate of follicular atresia increases, accelerating loss. This hypothesis was derived from old morphometric studies using log-linear plots of follicle number by age which demonstrate a deviation in the scatter of points at this approximate age ^11– 13^. Modern reassessment of the morphometric data has suggested that this deviation does not represent a shift to accelerated follicular loss but is in fact a distortion of the values caused by transformation of the data to a log-linear model ^14^. Hansen *et al*. used modern stereology techniques to count the number of non-growing follicles from 122 specimens and proposed that a constant increasing rate of follicular depletion occurs over the reproductive lifespan ^7^. These morphometric techniques are limited by availability of suitable tissue specimens and the difficult nature of counting primordial follicles.

Anti-Müllerian hormone (AMH) is secreted by the granulosa cells of antral and preantral follicles and therefore is reflective of the size of the primordial follicle pool. AMH may emerge as a prediction marker for menopause. Depmann *et al*. reported that AMH was an independent predictor of time to menopause but that the predictive effect became less strong with increasing subject age ^15, 16^. Larger cohort studies are yet to determine the utility of this tool and may also shed light on the pattern of primordial follicle loss occurring in the reproductive years preceding menopause ^15, 16^.

Subtle endocrinological changes to the menstrual cycle take place long before the shortening of the cycle and irregularity which heralds the onset of the perimenopause. In normal menstrual cycles, elevated FSH concentrations in the luteal-follicular transition enable the recruitment of gonadotrophin-dependent antral follicles and induce estradiol, inhibin A and inhibin B synthesis. In the early menopausal transition, inhibin B secretion by the relatively diminished antral follicle pool results in further increase in early follicular FSH. At the ovarian level, the increase in early follicular FSH results in earlier selection and growth of the dominant follicle and a relatively shorter follicular phase. Raised early follicular FSH and estradiol concentrations may also precipitate the development of multiple dominant follicles and the increase in dizygotic twin pregnancies in women of advanced maternal age. As the menopausal transition progresses, symptomatic with periods of amenorrhoea, estradiol and inhibin A decrease, contributing to the loss of negative ovarian feedback within the hypothalamic pituitary unit ^17^.

Oocyte quality appears to relate to the decline in the quality of oocyte cytoplasm and increasing nuclear genome abnormalities occurring with age ^18, 19^. The oocyte completes its first meiotic division at the time of ovulation induced by the midcycle surge in luteinising hormone (LH). The second meiotic division is completed at the time of fertilisation and the second polar body subsequently extruded. It is believed that age-related changes in the meiotic spindle formation and chromosome alignment result in the high rates of oocyte embryo aneuploidy in women of advancing reproductive age. Furthermore, age-related instability in mitochondrial DNA may affect oocyte competence and transmission of mitochondrial abnormalities to the offspring. Lower mitochondrial DNA and indeed mitochondrial content are predictive of poor oocyte quality and result in abnormal, premature mitochondrial biogenesis in early embryonic development ^19^.

Other meiotic cellular activities implicated in declining oocyte quality include telomere shortening and cohesion dysfunction. The cohesin protein complex maintains the cohesion of the sister chromatid structure. Cohesion cleavage is required for correct chromosomal segregation for meiosis. It is proposed that increasing time to resumption of meiosis in women of advancing age results in deterioration in cohesion and increasing segregation errors and aneuploidy. Increased separase activity, premature Shugoshin 2 degradation, activity of the anaphase-promoting complex or cyclosome and the spindle assembly checkpoint and oxidative stress are factors implicated in this failure ^20^.

Telomeres cap and protect the chromosome ends. This capping prevents the exposure of the DNA end which resembles a DNA break and prompts a DNA damage response. Telomere shortening and eventual exposure of the DNA end are inevitable consequences of ongoing cell division and increasing age. Uncapping of the chromosome ends may therefore clarify the pathogenesis of the multitude of age-related effects on oocyte cellular function, including cohesion defects, disrupted chiasma and meitotic spindle, aneuploidy and reactive oxygen species (ROS) damage. Keefe *et al*. ^21– 23^ have proposed that the shortening of oocyte telomeres associated with increasing oocyte fragmentation and aneuploidy is an evolutionary mechanism to prevent women of advanced maternal age from carrying a pregnancy that would risk their own health ^24^.

Much of what is understood regarding oocyte quality is the result of evaluation of oocytes and granulosa cells collected from periovulatory follicles in the context of gonadotrophin stimulation. In women more than 38 years old, the luteinising granulosa cells are less numerous, produce less steroids and glycoproteins and display more mitochondrial damage and mitochondrial DNA deletions. These follicles show less expression of antioxidant enzymes which may contribute to an imbalance of oxidant and antioxidant systems leading to oxidative stress, implicated in subfertility. The timing and mechanism by which these changes arise are poorly understood ^25^.

The age at which the final menopausal transition occurs is multifactorial. Women who experience earlier menopause will accordingly experience earlier age-related sterility ^10^. There appears to be a strong genetic component to the age of menopausal onset, and associations in menopausal age are demonstrated in mother–daughter and sister pairs ^26^.

Genes that exert an effect on reproductive hormones, follicular function and folliculogenesis include FSH beta, FSH receptor, LH beta, LH receptor, 17 α hydroxylase/17, 20 (CYP17) and aromatase (CYP 19). Bone morphogenic protein 15 (BMP 15), growth differentiation factor 9 (GDF 9) and G protein receptor 3 (GPR 3) have a role in determining the rate of follicular recruitment. Both groups have been implicated in the pathogenesis of some cases of premature ovarian insufficiency. Therefore, it is possible that pleomorphisms in these or other genes contribute to variation in the reproductive lifespan ^27^.

Many studies have investigated the impact of lifestyle choices on reproductive ageing and the data are conflicting. These factors—including tobacco use and smoking, combined oral contraceptive pill (COCP) use, body mass index (BMI) and alcohol use—may be correlated with the age of spontaneous menopause. Morphological studies have reported conflicting data in this area. Westhoff *et al*. examined the ovaries of 89 premenopausal women ^28^. Follicle density was significantly lower in ever smokers as compared with never smokers and in ever alcohol users as compared with never users. These associations were significant after controlling for age. There was no association demonstrated between follicle density and COCP or BMI ^28^. A similar study has since examined the follicle count in entire premenopausal ovary specimens ^6^. Cumulative alcohol use was positively associated with ovarian follicle count in that study; however, BMI, COCP use and smoking showed no association ^6^.

## Conclusions

Decline in oocyte quantity is influenced by the rate of follicular depletion and size of the initial follicle pool. The reductions in oocyte quantity and oocyte quality inherent in reproductive ageing result in clinical subfertility and increased miscarriage rates in women of advanced maternal age. An improved understanding of the mechanisms in involved in female reproductive ageing may result in the development of biomarkers to predict the timing of age-related declining fertility in an individual and provide target points for therapeutics.
